# Transformer-Based High-Frequency Oscillation Signal Detection on Magnetoencephalography From Epileptic Patients

**DOI:** 10.3389/fmolb.2022.822810

**Published:** 2022-03-04

**Authors:** Jiayang Guo, Naian Xiao, Hailong Li, Lili He, Qiyuan Li, Ting Wu, Xiaonan He, Peizhi Chen, Duo Chen, Jing Xiang, Xueping Peng

**Affiliations:** ^1^ Department of Hematology, The First Affiliated Hospital of Xiamen University and Institute of Hematology, School of Medicine, Xiamen University, Xiamen, China; ^2^ Department of Neurology, The First Affiliated Hospital of Xiamen University, Xiamen, China; ^3^ Department of Radiology, Imaging Research Center, Cincinnati Children’s Hospital Medical Center, Cincinnati, OH, United States; ^4^ Department of Radiology, Jiangsu Province Hospital of Chinese Medicine, Affiliated Hospital of Nanjing University of Chinese Medicine, Nanjing, China; ^5^ Emergency Critical Care Center, Beijing Anzhen Hospital, Capital Medical University, Beijing, China; ^6^ College of Computer and Information Engineering, Xiamen University of Technology, Xiamen, China; ^7^ School of Artificial Intelligence and Information Technology, Nanjing University of Chinese Medicine, Nanjing, China; ^8^ Department of Neurology, The MEG Center, Cincinnati Children’s Hospital Medical Center, Cincinnati, OH, United States; ^9^ Australian AI Institute, FEIT, University of Technology Sydney, Sydney, NSW, Australia

**Keywords:** magnetoencephalography, high-frequency oscillation, transformer, deep learning, epilepsy

## Abstract

High-frequency oscillations (HFOs), observed within 80–500 Hz of magnetoencephalography (MEG) data, are putative biomarkers to localize epileptogenic zones that are critical for the success of surgical epilepsy treatment. It is crucial to accurately detect HFOs for improving the surgical outcome of patients with epilepsy. However, in clinical practices, detecting HFOs in MEG signals mainly depends on visual inspection by clinicians, which is very time-consuming, labor-intensive, subjective, and error-prone. To accurately and automatically detect HFOs, machine learning approaches have been developed and have demonstrated the promising results of automated HFO detection. More recently, the transformer-based model has attracted wide attention and achieved state-of-the-art performance on many machine learning tasks. In this paper, we are investigating the suitability of transformer-based models on the detection of HFOs. Specifically, we propose a transformer-based HFO detection framework for biomedical MEG one-dimensional signal data. For signal classification, we develop a transformer-based HFO (TransHFO) classification model. Then, we investigate the relationship between depth of deep learning models and classification performance. The experimental results show that the proposed framework outperforms the state-of-the-art HFO classifiers, increasing classification accuracy by 7%. Furthermore, we find that shallow TransHFO ( < 10 layers) outperforms deep TransHFO models (≥10 layers) on most data augmented factors.

## 1 Introduction

It is estimated that about 1% of the population around the world is affected by epilepsy (Health Quality Ontario, 2012). Long-term follow-up studies in epilepsy indicate that approximately 30% of epilepsy cases are intractable to medical therapy (Yang et al., 2021), but may benefit from surgery (Ibrahim et al., 2012; Zhang et al., 2019; Guo et al., 2021). A favorable surgical outcome depends on many factors, one of which is the accurate identification of epileptogenic zones (Rosenow and Lüders, 2001). Magnetoencephalography (MEG) is a non-invasive technology for pre-operative workup prior to epilepsy surgery (Xiang et al., 2009), which help clinicians localize epileptogenic zones (Rampp et al., 2010; Niranjan et al., 2013). Recently, several studies showed that high-frequency oscillations (HFOs), which can be observed within 80–500 Hz of MEG data, are putative biomarkers to locate the epileptogenic tissue. It has the potential to improve the presurgical diagnosis and surgical outcome of patients with epilepsy (von Ellenrieder et al., 2016; Van Klink et al., 2016; Papadelis et al., 2016).

In current clinical practices, machine learning methods are widely used for clinical classification problems using one-dimensional biomedical signal data (e.g., MEG, EEG) (Fernandez-Blanco et al., 2020; Li et al., 2020; Maiorana, 2020; Lombardi et al., 2021). Detecting HFOs in MEG signals mainly depends on visual inspection by surgeons. Such visual identification of HFOs is very time-consuming, labor-intensive, subjective, and error-prone due to the short duration and low amplitude of HFOs and the large volume of MEG signal data (Zelmann et al., 2012). Thus, helping clinicians with HFO detection can be treated as a clinical classification problem. To detect HFOs accurately and automatically, machine learning approaches have been used (Elahian et al., 2017; Guo et al., 2018; Weiss et al., 2019). Most existing HFO detection models the first segment a fixed length of MEG signals from the whole MEG data, then treat these MEG signal segments as feature vectors. For example, one of the earlier works on HFO detection models employed the fully connected feed-forward network to automatically learn the distribution of segmented MEG signals (Guo et al., 2018). These studies have demonstrated that machine learning models were able to achieve promising results on automatic identification of HFO signals.

More recently, a novel model architecture, called Transformer, has attracted wide attention in natural language processing and computer vision (Vaswani et al., 2017; Zhai et al., 2021). The Transformer utilized a self-attention mechanism to learn an input feature sequence and decide which parts of the sequence are important. It has outperformed peer models and achieved state-of-the-art performance on many machine learning tasks, such as language translation (Vaswani et al., 2017), image classification (Dosovitskiy et al., 2020), speech recognition (Kim et al., 2020), time-series data processing (Li et al., 2019), and healthcare analytics (Peng et al., 2019; Peng et al., 2020; Peng et al., 2021). Due to the fact that MEG signals are inherently time-series signal data, we are wondering whether the Transformer model is able to understand the time dependency embedded in the MEG signals better than existing fully connected feed-forward network-based models.

In this paper, we are investigating the suitability of the Transformer model on the detection of HFOs from MEG data. Furthermore, we are questioning what the preferred architecture of the Transformer-based deep learning model is for MEG signal classification tasks. We propose a Transformer-based HFO detection framework designed for one-dimensional biomedical MEG signal data. Briefly, the framework includes signal segmentation, virtual sample generation, signal classification, and signal labeling. Within this framework, we developed and validated a Transformer-based HFO (TransHFO) classification model to distinguish HFO signals from normal signals. We designed experiments to investigate whether the TransHFO model is able to achieve robust and reliable performance on HFO classification. Furthermore, with the TransHFO classification framework, we set to conduct exploratory experiments to test if the relationship between the depth of TransHFO models and classification performance is positively monotonic. Namely, we would investigate whether HFO classification performance would increase as the depth of TransHFO model increases. This would guide the transformer-based model design on HFO classification problems, as well as other similar tasks using one-dimensional biomedical signal data (e.g., MEG, EEG).

To summarize, our main contributions are as follows:

• We proposed a novel and effective transformer-based HFO detection framework designed specifically for the presurgical diagnosis of biomedical one-dimensional MEG signal data.
• We investigated through quantitative experiments whether transformer-based deep learning models are able to achieve robust and reliable performance on HFO classification task.
• We conducted exploratory experiments to examine the relationship between the depth of transformer-based deep learning models and the classification performance, which would result in principles and insights for future model design.

The remainder of this paper is organized as follows: Section 2 briefly reviews the related work on MEG data. Section 3 describes the proposed detection framework. Section 4 presents the experiments and results on the real-world MEG dataset, and Section 5 concludes the paper by summarizing the research and presenting future directions.

## 2 Related Work

In this section, we briefly reviewed existing works of machine learning-based approaches for automated identification of the epileptic HFOs and Transformer-based detector on clinical applications.

Machine learning provides clinicians and surgeons with a possible opportunity for improving the performance of detecting HFOs and reducing human interference. Traditional machine learning algorithms, such as logistic regression (Elahian et al., 2017), have been used for the identification of epileptogenic zones. Deep neural network-based models also have been exploited to detect HFOs in MEG signal data. This includes our prior work using an auto-encoder-based SMO detector (Guo et al., 2018). In another study, Weiss et al. (2019) discuss possible machine learning strategies that can be applied to HFOs to better identify epileptogenic regions. It set to apply a virtual sample generation approach to increase the size of training samples for the deep learning model. Here, we utilize an adaptive synthetic (ADASYN)-based virtual sample generation approach for our MEG dataset (He et al., 2008).

More recently, Transformer-based approaches are the current state of the art in many clinical tasks, such as clinical text classification (Gao et al., 2021) and predicting depression (Meng et al., 2021). The key component of the Transformer is the multi-head attention mechanism, which can avoid information loss over time steps compared to recurrent structures. Research has proposed the integration of attention mechanism and convolutional neural networks (CNN) to classify categorized images from visual evoked MEG brain signals (Kim et al., 2019). So far, these approaches have shown promising prediction accuracy, but some argue that the power of attention mechanism in a CNN is limited by the weaknesses of the CNN. Vaswani et al. (2017) used a sole attention mechanism to construct a sequence-to-sequence model for a neural machine translation task that achieved a state-of-the-art quality score. According to Shen et al. (2018), attention mechanism allows for more flexibility, and is more task/data-driven when modeling dependencies. Unlike sequential models, attention mechanism is easy to compute. Computation can also be significantly accelerated with distributed/parallel computing schemes. However, to the best of our knowledge, a model based entirely on Transformer structure has not yet been designed for analysis of MEG data.

## 3 Transformer-Based HFO Detection Framework

This section begins with introducing the overview of HFO detection framework. Then, we described virtual sample generation. Next, the TransHFO classification model with the dense layer and transformer model for biomedical MEG one-dimensional signal data is elaborated.

### 3.1 Overview of HFO Detection Framework

**FIGURE 1:**
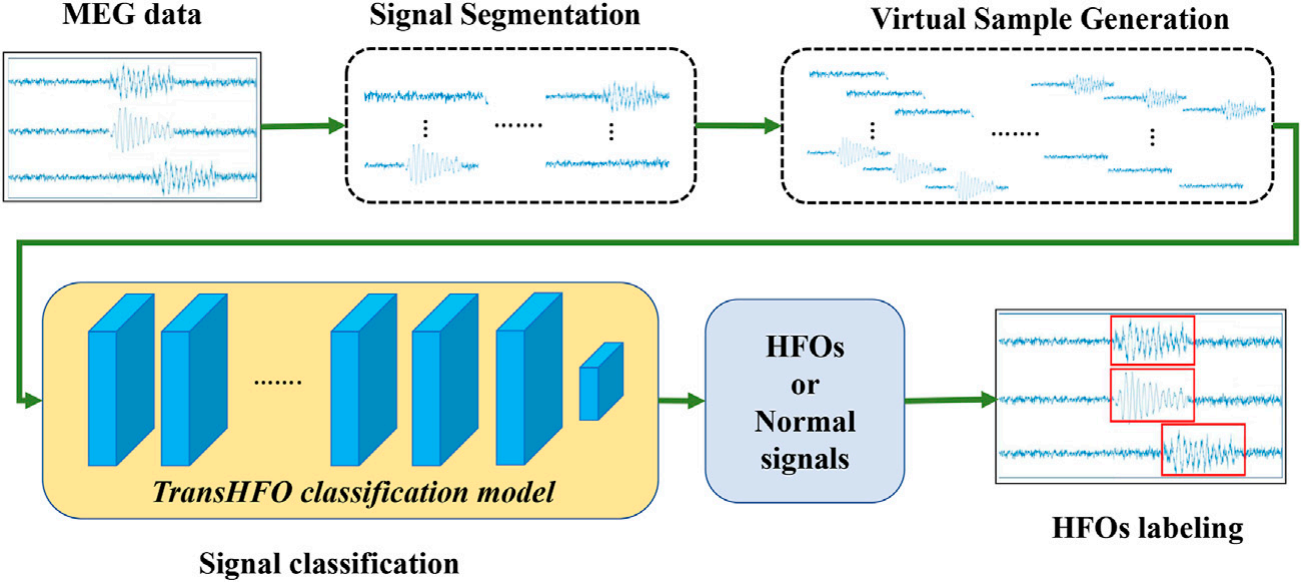
The proposed Transformer-based HFO detection framework is designed specifically for the presurgical diagnosis of biomedical one-dimensional MEG signal data. Briefly, the HFO classification framework includes signal segmentation, signal augmentation, TransHFO signal classification, and signal labeling. This framework achieves more robust and reliable performance on HFO classification than baseline models. Furthermore, we find that shallow TransHFO ( < 10 layers) outperforms deep TransHFO (≥10 layers) on most data augmented factors, revealing the importance of human labeled data and the potential of deep-learning methods for automatic diagnosis of medical signal.

The overview of transformer-based HFO detection framework is described in Figure 1. Specifically, the framework includes signal segmentation, virtual sample generation, signal classification, and signal labeling. We developed a TransHFO model for HFO signal classification. The framework contains a training phase and a testing phase. During the training phase, given the gold standard of MEG signal segments with a certain duration (i.e., 1,000 ms), the virtual sample generation method is used to augment the size of the HFOs and normal control (NC) signals. A TransHFO model is trained to distinguish HFOs from NC signals. During the testing process, given a set of MEG data, the framework split the data into a series of signal segments with a moving window into the same length of training data. Then, the trained TransHFO model is used to classify the segments. The assigned labels can be visualized by software such as the MEG processor (Xiang et al., 2015).

### 3.2 Virtual Sample Generation

To increase the size of training samples for the deep learning model, a virtual sample generation approach has been applied. Here, an adaptive synthetic (ADASYN)-based virtual sample generation approach is utilized for our MEG dataset (He et al., 2008). ADASYN was originally proposed to perform over-sampling for imbalanced datasets. However, it has also been applied to increase sample size when the training samples of machine learning models are insufficient (Kawahara et al., 2017). The ADASYN approach first calculates the degree of imbalance between minority and majority class samples. If the degree of imbalance is smaller than a preset threshold for maximum tolerated imbalance, it estimates the number of virtual samples to be generated from the minority class. For each sample in the minority class, this approach finds the k-nearest neighbors (KNN) based on Euclidean distance and calculates the density distribution of the minority class for the given minor sample. Eventually, it generates virtual samples for each minority sample based on estimated sample size and density distribution.

To utilize the ADASYN as the virtual sample generation method for our balanced MEG dataset, we manually create an imbalanced dataset from our ground truth data. Specifically, both HFOs and normal control (NC) samples are separated into three bins, respectively. One bin of HFO samples and three bins of NC samples are combined as an imbalanced dataset. The ADASYN is then applied to generate virtual ripple samples for this temporary imbalanced dataset. Similarly, the ADASYN is utilized to synthesize NC samples. This procedure is repeated until the pre-defined number of virtual samples is generated. By varying different data augmented factors, we conducted exploratory experiments to examine the relationship between the depth of deep learning models and the classification performance, resulting in several principles and insights for future model design [t].

### 3.3 TransHFO Classification Model

**FIGURE 2:**
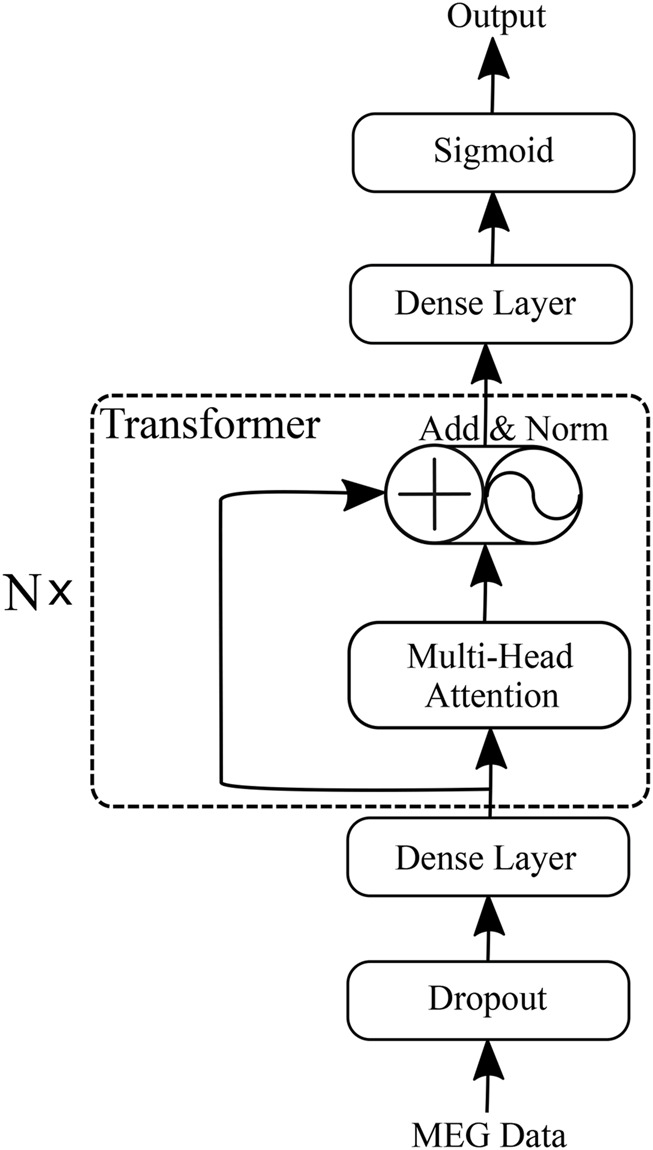
Transformer-based HFO (TransHFO) classification model.

We propose an HFO classification framework called the “Transformer-based HFO (TransHFO)” classification. The architecture of TransHFO is shown in Figure 2. The framework consists of a dropout layer, a stack of *N* identical layers, and two dense layers.

Each layer in *N* stacked transformer has two sub-layers (Vaswani et al., 2017). The first is a multi-head self-attention mechanism, and the second sub-layer is a feed-forward network. Residual connection (He et al., 2016) is employed around each of the two sub-layers, followed by layer normalization (Ba et al., 2016). That is, the output *o* of each layer is

$$o_{i}=Norm\left(ReLU\left(sublayer\left(o_{i-1}\right)+o_{i-1}\right)\right),$$ (1)

where *i* is the *i*th layer in transformer blocks and sublayer (***o***_*i*−1_) is the function implemented by the layer itself.

The two dense layers are fully connected to change the number of units in the framework. The dense layer (lower) and layer (upper) are set with activation “ReLU”, and 128 and 10 units of the output space, respectively. The third dense layer is “Sigmoid” in the framework with 1 unit of the output space and sigmoid activation function.

A standard cross-entropy loss is used as the training objective of TransHFO, defined as

$$L=-\overset{k}{\underset{i=1}{\sum }}y_{i}\log\left({\hat{y}}_{i}\right),$$ (2)

where *y* is the one-hot target for medical outcome and ${\hat{y}}_{i}$ is the probability of the *i*th class given patient journey.

#### 3.3.1 Dense Layer

Dense layer can be thought of exploring the importance of each signal within a sequence and compresses the sequence of signals into a low-dimension vector representation. For simplicity, we take the dropout output ***s*** as an example. Formally, it is written as:

$$o=w^{T}\sigma \left(w^{\left(1\right)}s+b^{\left(1\right)}\right)+b,$$ (3)

where *σ* is ReLU function and ***w***, ***w*** ^(1)^, ***b*** ^(1)^, ***o*** are learnable parameters.

#### 3.3.2 Transformer

To learn relationships between patients’ MEG, the Transformer module is proposed to capture the inherent dependencies, which is calculated as follows:

v=Transformer(o) (4)

The Transformer is identical to that of BERT (Vaswani et al., 2017) and (Devlin et al., 2018), which has two sub-layers. The first is a multi-head attention mechanism (explained below), and the second is a position-wise addition and normalization layer. A residual connection (He et al., 2016) is employed around each of the two sub-layers, followed by layer normalization (Ba et al., 2016).

The multi-head attention mechanism relies on self-attention, where all of the keys, values, and queries come from the same place. The self-attention operates on a query ***Q***, a key ***K***, and a value ***V***:

$$Attention\left(Q,K,V\right)=softmax\left(\frac{QK^{T}}{\sqrt{d}}\right)V$$ (5)

where ***Q***, ***K***, and ***V*** are *n* × *d* matrices, *n* denotes the number of diagnoses in a visit in a patient record, and *d* denotes the embedding dimension.

The multi-head attention mechanism obtains *h* (i.e., one per head) different representations of (***Q***, ***K***, ***V***), computes self-attention for each representation, and concatenates the results. This can be expressed as follows:

$${head}_{i}=Attention\left(QW_{i}^{Q},KW_{i}^{K},VW_{i}^{V}\right)$$ (6)

$$MultiHead\left(Q,K,V\right)=Concat\left({head}_{1},\ldots ,{head}_{h}\right)W^{O}$$ (7)

where the projections are parameter matrices $W_{i}^{Q}\in R^{d\times d_{k}}$ , $W_{i}^{K}\in R^{d\times d_{k}}$ , $W_{i}^{V}\in R^{d\times d_{v}}$ and $W^{O}\in R^{hd_{v}\times d}$ , *d*_*k*_ = *d*_*v*_ = *d*/*h*.

## 4 Experiments

We conducted experiments based on a real-world MEG dataset to compare the performance of our proposed method TransHFO with several state-of-the-art methods in terms of the classification performance. We also evaluated the impact of the data augmentation on the baseline methods and our TransHFO model. Furthermore, we explored the influence of the network depth on TransHFO model.

### 4.1 Dataset Description

MEG data were obtained from 20 clinical patients (age: 6–60 years, mean age 32; 10 female patients and 10 male patients) affected by localization-related epilepsy, which is characterized by partial seizures arising from one part of the brain, and were retrospectively studied. The data were acquired under approval from an Institutional Review Board. MEG recordings were performed in a magnetically shielded room (MSR) using a 306-channel, whole-head MEG system (VectorView, Elekta Neuromag, Helsinki, Finland). The sampling rate of MEG data was set to 2,400 Hz, and approximately 60 min of MEG data were recorded for each patient. MEG data were preliminarily analyzed at a sensor level with MEG Processor (Xiang et al., 2015; Li and Yin, 2020). The spike was visually identified in waveform with a band-pass filter of 1–70 Hz, while HFOs were analyzed with a band-pass filter of 80–500 Hz. For the model evaluation purpose, the clinical epileptologists selected HFOs and NC signal segments based on intracranial recordings (iEEG) for these patients. By comparing the MEG sources and the brain areas generating HFOs, the clinical epileptologists marked HFOs. The duration of each signal segment which contains a series of 2000 signal time points is 1 s. A total of 202 signal segments (101 HFO samples and 101 NC samples) were composed as a gold standard dataset for model evaluation.

### 4.2 Experiment Setup

#### 4.2.1 Model Evaluation

We conducted a comprehensive evaluation in this study by employing the proposed TransHFO to classify the HFO signals from normal controls. A *k*-fold cross-validation was designed in our experiments. The whole gold standard dataset would be divided into *k* portions. In each repeated iteration, we randomly used one portion of the data as testing data, and applied the rest (*k*-1) portions of the data as training data. This process would be repeated *k* times until all data have been tested once. The classification performance was evaluated by aggregating all iterations.

#### 4.2.2 Baseline Methods

We choose three baseline methods: Logistic regression, which is traditional machine learning model; SMO (Guo et al., 2018), which is the latest deep learning model used in MEG data; and the ResDen model, which is the simplified version of our proposed TransHFO model.

• Logistic regression (LR): The maximum-likelihood estimation algorithm was used to optimize the coefficient of the logistic regression model.
• SMO: We implemented 4-layer SSAE-based neural networks with an input layer, three hidden layers, and an output layer. The number of nodes in three hidden layers was set to 30. A loss function with L2 regularization and sparsity regularization terms were utilized. Hyper parameter sparsity proportion was selected from [0.1, 0.2, 0.3, 0.4, 0.5] and L2 regularization weight was decided from [0.1, 0.2, 0.3, 0.4, 0.5]. The learning rate was set to 0.01. The training is stopped if the model returns the same loss on validation data in three consecutive epochs.
• ResDen: ResDen follows the same framework as TransHFO, but Multi-Head Attention is replaced with a simple dense layer with “ReLU” activation and having the same number of units as that of Dense-1 in the TransHFO model.

#### 4.2.3 Evaluation Metrics

We calculated true positive (TP), false positive (FP), true negative (TN), and false negative (FN) for the classification by comparing the classified labels and gold-standard labels. Then, we calculated accuracy, sensitivity, precision, and F-score by:

$$\begin{array}{ll} & Accuracy=\frac{TP+TN}{TP+TN+FP+FN}\\ & Precision=\frac{TP}{TP+FP}\\ & Sensitivity=\frac{TP}{TP+FN}\\ & Specificity=\frac{TN}{TN+FP}\\ & F-score=2\times \frac{precision\times sensitivity}{precision+sensitivity} \end{array}$$ (8)

#### 4.2.4 Implementation Details

We implement all the approaches with Tensorflow 2.0, except LR. For training models, we use RMSprop with a mini-batch of 32 patients and 10 epochs. The drop-out rate is 0.1 for all the approaches. Virtual samples are generated by ADASYN, and the size of samples is the scalars (1, 5, 10, 20, and 40) times the size of the original data. The number of the stacked *N* identical layers varies from 1 to 40 in the TransHFO framework.

### 4.3 Results

#### 4.3.1 HFO Performance Comparison Using Different Models

In this section, we first compared HFO classification performance of the proposed framework with the-state-of-art models. As shown in Table 1, we set up two scenarios: with data augmentation and without data augmentation. The upper part of Table 1 illustrated HFO performance using only gold standard data without any data augmentation scheme. In our experiments, traditional machine learning model LR had the lowest performance compared to other three neural network-based models. The proposed model achieved the best performance with an accuracy of 0.9580, a precision of 0.9929, a sensitivity of 0.9289, a specificity of 0.9917, and an F-score of 0.9593. It achieved better performance than our previously developed SMO detector. Compared to ResDen, our model had higher accuracy, precision, specificity, and F-score, while both our model and ResDen reached 0.9289 on sensitivity.

**TABLE 1:** Performance comparison of different models. The TransHFO achieved better performance than LR, SMO, and ResDen models in both no data augmentation and data augmentation scenarios.

Model	Accuracy	Precision	Sensitivity	Specificity	F-score
LR	0.807 7	0.909 1	0.714 3	0.916 7	0.800 0
SMO	0.846 2	0.916 7	0.785 7	0.916 7	0.846 2
ResDen	0.923 1	0.928 6	0.928 6	0.916 7	0.928 6
TransHFO	**0.958 0**	**0.992 9**	**0.928 9**	**0.991 7**	**0.959 3**
LR (Aug)	0.807 7	0.909 1	0.714 3	0.916 7	0.800 0
SMO(Aug)	0.884 6	0.923 1	0.857 1	0.916 7	0.888 9
ResDen (Aug)	0.948 7	0.952 4	0.952 4	0.944 4	0.952 4
TransHFO(Aug)	**0.961 5**	**1.000 0**	**0.928 6**	**1.000 0**	**0.963 0**

Bold values represents the best performance of different models in the corresponding Evaluation Metrics

For the data augmentation scenario, the proposed model had an accuracy of 0.9615, a precision of 1.0, a sensitivity of 0.9286, a specificity of 1.0, and an F-score of 0.9630. This is again the best among four compared models. Also, the proposed model and ResDen also had better performance than the LR model and SMO detector, demonstrating the superior strength of deep learning models. Compared to ResDen, our model achieved better performance on accuracy, precision, specificity, and F-score. We believe that this is due to the multi-head attention mechanism added into the model. Furthermore, by comparing the upper part and lower part of the table, we noted that, except LR, these three neural network-based models all had improved performance with data augmentation, illustrating the effectiveness of data augmentation on neural networks. For SMO and ResDen, the accuracy increased 4% and 2%, respectively. However, the proposed TransHFO model only had a slight increase on accuracy (0.5%). The effect of data augmentation is very limited.

#### 4.3.2 Impact of Data Augmentation

**FIGURE 3:**
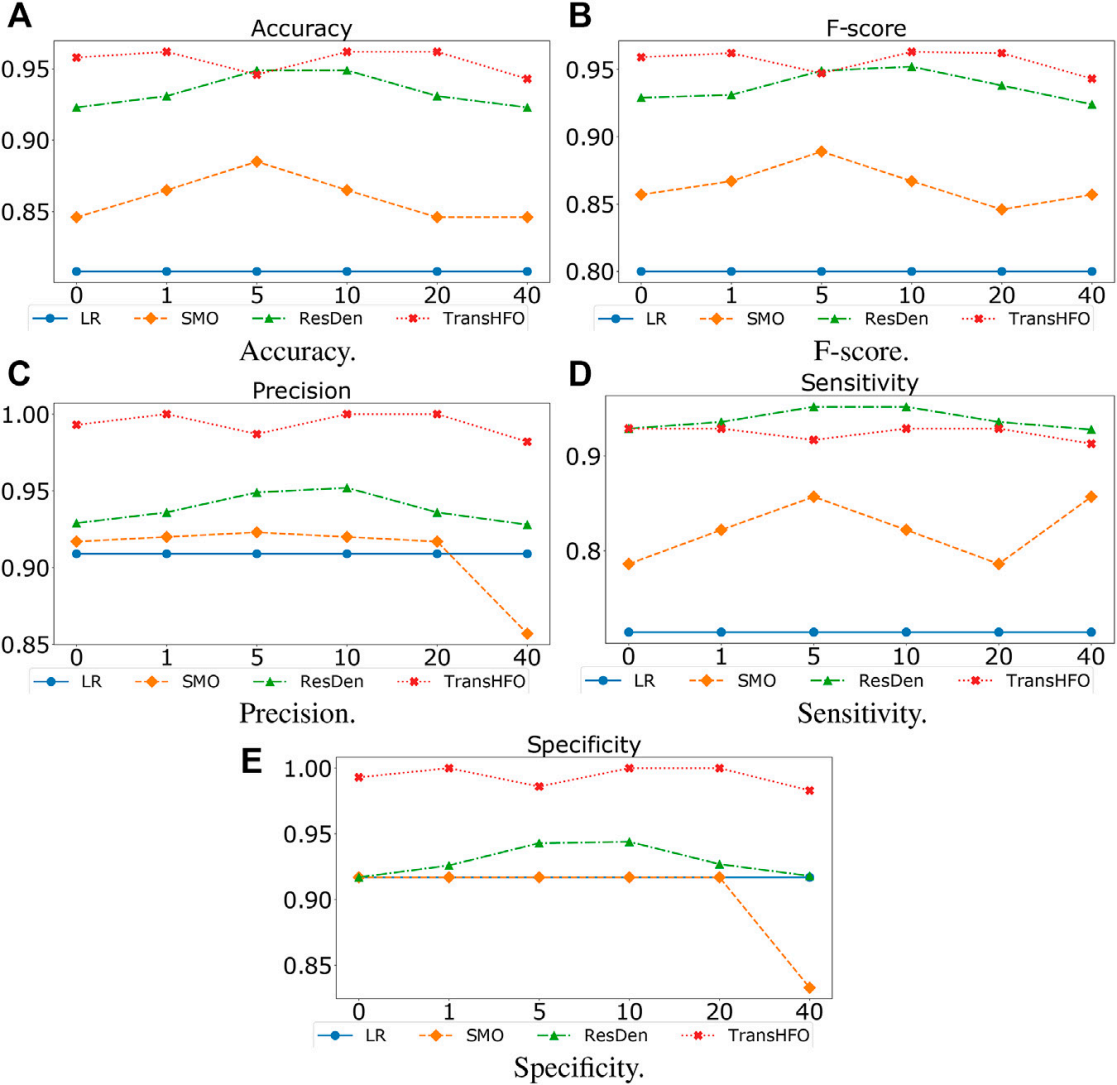
Performance of different models with varying augmentation factors from 0 to 40. The models had better performance with augmentation factors 5, 10, and 20. **(A)** Accuracy. **(B)** F-score. **(C)** Precision. **(D)** Sensitivity. **(E)** Specificity.

Figure 3 listed the HFO classification performance of the proposed TransHFO model as well as the other three models, tested on dataset augmented by 0- to 40-fold. The LR model has no performance change using different augmented data. For TransHFO, the best accuracy and F-score were 0.9615 and 0.963, respectively, at an augmentation factor of 10. For both ResDen and SMO, the best performance was obtained by using an augmentation factor of 5. A clear trend showed that all neural network-based models achieved improved performance with an augmentation factor of 5 or 10. However, the incremental changes of performance on accuracy and F-score were limited. Combined with previous experiment, we believe that data augmentation is a technique for increasing sample data so as to avoid model overfitting. This will prevent the model achieved overfitted low performance. On the other hand, data augmentation may not be effective to increase the performance of HFO classification. Considering the training time cost, an augmentation factor that is able to increase the sample size to 1,000–2,000 may be sufficient for training a model with residual links and attention mechanism.

#### 4.3.3 Impact of Network Depth of TransHFO

**FIGURE 4:**
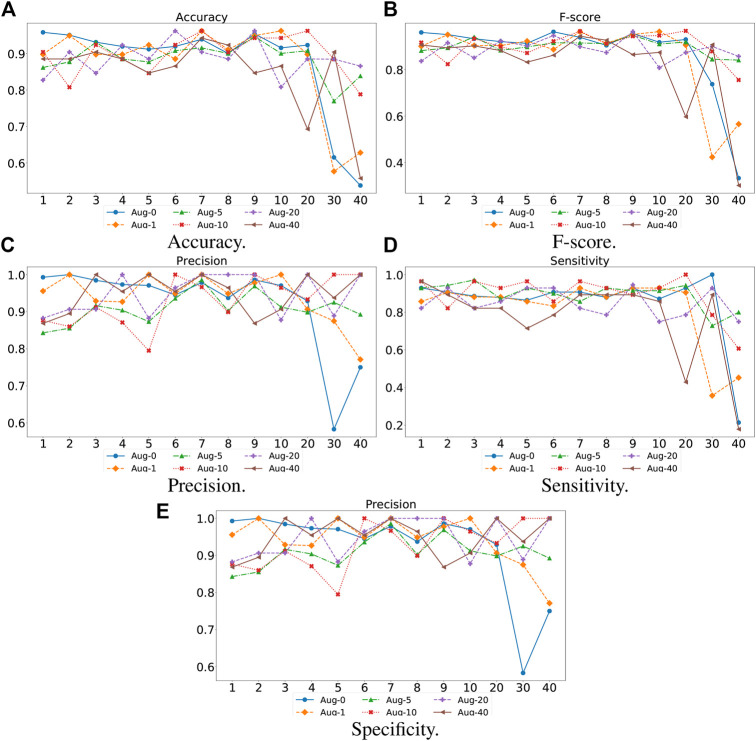
Performance of different augmentation factors (0, 1, 5, 10, 20, and 40) with varying *N*, the number of identical layers in TransHFO framework, from 1 to 40. **(A)** Accuracy. **(B)** F-score. **(C)** Precision. **(D)** Sensitivity. **(E)** Specificity.

For the proposed TransHFO, we tested the HFO classification performance using different architecture and augmentation factors. As displayed in Figure 4, we listed the accuracy, F-score, precision, sensitivity, and specificity, respectively. First, a general trend of these figures showed that the HFO classification performance decreased dramatically when the model had more than 10 layers. If we use 10 layers as a cutoff between shallow TransHFO ( < 10 layers) and deep TransHFO (≥10 layers), the results demonstrated that the shallow TransHFO achieved better HFO classification performance than deep TransHFO. For the one-dimensional biomedical MEG signals, the more layers of the model may not increase the performance. This observation needs further confirmation by using additional experiments with more bio-signals.

Second, we noted that when the training data were augmented by a factor of 10, the proposed TransHFO achieved the best performance. However, as mentioned in the previous section, the performance of HFO classification was quite similar for shallow TransHFO using very different augmentation factors from 0 to 40. On the other side, the performance of HFO classification had very large variations for deep TransHFO using different augmented training data. The results suggest that for bio-signal MEG signals, a small augmentation factor and a shallow TransHFO model would be efficient to achieve a desirable performance. Additional data augmentation or deeper architecture may not improve the performance of HFO classification.

### 4.4 Discussion

HFO classification task is a crucial step towards surgical treatment on epilepsy patients. With the ground truth training dataset, the TransHFO detector achieves an accuracy of 96.15*%* on the HFO classification task. This sheds light on the feasibility of using transformer-based networks techniques. Meanwhile, we find that shallow TransHFO ( < 10 layers) outperforms deep TransHFO models (≥10 layers) on most HFO classification tasks with different data augmented factors. This finding offers us the possibility to use interpretable and shallow models based on appropriately expressing the structure of MEG data for precise HFO detection.

This study mainly focuses on the shallow transformer-based model that can achieve better performance than the deep learning model in HFO detection tasks. Supported by the results, the TransHFO detector benefits from both the virtual sample generation technique and the multi-head attention mechanism. However, according to Figure 4, the shallow TransHFO achieved better performance than deep TransHFO in HFO classification tasks with different data augmented factors. For the HFO detection task, more layers of the model may decrease the performance. The strengths of shallow TransHFO over deep TransHFO in the HFO classification tasks may partially be attributed to the nature of the dataset. The dataset is a small dataset that consists of one-dimensional biomedical MEG signals from one institution. For such a dataset, the virtual sample generation technique may not augment enough higher-level features of MEG signals that can be learned by deep TransHFO. Additionally, our observation needs further confirmation by using additional experiments with more bio-signals from external data sites.

There are several limitations in this study. First, we have a small cohort of epilepsy patients. We only collect 202 samples from 20 patients. Although the virtual sample generation approach mitigates the insufficient issue in a way, a larger cohort possibly provides better biological varieties. Second, according to clinical routine, we pre-defined the duration of MEG signal segments as 1 s. Additional information for HFO classification may be revealed by other signal duration (e.g., 0.5–2 s). Third, this paper treats the MEG segments from different channels equally and independently. However, there are complex timing and co-occurrence relationships among segments. Mining and utilizing these relationships may improve the effectiveness of HFO detection. Finally, only internal validation was conducted on a set of data from one institution. Additional datasets from external data sites are required to test the generalizability of our detector.

## 5 Conclusion

In this paper, we presented a novel Transformer-based HFO detection framework designed specifically for biomedical MEG one-dimensional signal data. The proposed HFO detection framework employs Transformer models with a self-attention mechanism and virtual sample generation technique. Compared with the previously developed HFO classifiers, the proposed framework increased classification accuracy by 7%. With this new framework, we designed experiments to investigate whether deep TransHFO models are able to achieve robust and reliable performance on HFO classification. Furthermore, with the proposed classification framework, we set to conduct exploratory experiments to discover whether the relationship between the depth of TransHFO models and classification performance is positively monotonic. Based on exploratory experiments, we find that shallow TransHFO ( < 10 layers) outperforms deep TransHFO (≥10 layers) on most data augmented factors. The experimental results demonstrate that our proposed framework is a promising computer-aided diagnosis tool for clinical usage. Several future directions are clarified as follows. The first is to extend our framework into a multi-label classifier with a function to recognize additional patterns or sub-patterns (e.g., spike, ripple and fast ripple) in MEG. The second direction includes external validation and clinical applications of the proposed framework on other neuromagnetic data such as iEEG. The final direction is to design an interpretable and shallow network based on appropriately expressing the structure of MEG data for precise HFO detection.

## Data Availability

The data analyzed in this study is subject to the following licenses/restrictions: The raw data supporting the conclusions of this article will be made available by the authors, without undue reservation. Requests to access these datasets should be directed to corresponding author. Requests to access these datasets should be directed to wsxna@163.com.
